# Serial mediation of the relationship between impulsivity and suicidal ideation by depression and hopelessness in depressed patients

**DOI:** 10.1186/s12889-023-16378-0

**Published:** 2023-07-31

**Authors:** Xiaoli Chen, Shupeng Li

**Affiliations:** 1 School of Teacher Education, Weifang University, Shandong, China; 2 School of Economics and Management, Shandong Vocational College of Information Technology, Shandong, China

**Keywords:** Impulsivity, Depression, Hopelessness, Suicidal ideation, Mediation

## Abstract

**Background:**

Close relationships have been observed among impulsivity, depression, hopelessness, and suicidal ideation in depressed patients. However, the precise mechanism that connects these psychological symptoms remains unclear. This study aims to explore the mediation effect of depression and hopelessness on the relationship between impulsivity and suicidal ideation in depressed patients.

**Methods:**

A total of 258 depressed patients were evaluated using the Hamilton Depression Scale, the Beck Hopelessness Scale, the Scale for Suicide Ideation, and the Barratt Impulsiveness Scale. A path analysis was afterwards performed to determine the specified relationships in the proposed model.

**Results:**

The relationship between impulsivity and suicidal ideation was found to be serially mediated by depression and hopelessness. The mediating effect of depression and hopelessness accounted for 26.59% of the total effect. Specifically, in the pathway from impulsivity to hopelessness, the mediating effect of depression accounted for 40.26%. Moreover, the relationship between impulsivity and suicidal ideation was mediated by hopelessness, with the mediating effect accounting for 12.41%. It is important to note that these relationships were observed to be independent of age and marital status. Furthermore, the proposed model demonstrated a good fit with the data.

**Conclusions:**

This study identified a serial mediation pathway between impulsivity and suicidal ideation, mediated by depression and hopelessness. Our findings indicate that impulsivity indirectly influences suicidal ideation through its association with depression, which subsequently contributes to feelings of hopelessness. These results emphasize the importance of addressing symptoms of depression and hopelessness in the prevention and intervention efforts targeting individuals with depression. Additionally, monitoring and addressing impulsivity levels may also be crucial in reducing the risk of suicidal ideation among this population. These findings provide valuable insights for future preventive programs and interventions aimed at mitigating suicidal ideation in individuals with depression.

## Background

A body of research has consistently shown an association between suicidal ideation and major depressive disorder (MDD) [1, 2]. Suicidal ideation is prevalent among individuals with depression and is considered a precursor to suicide attempts and completed suicides [3]. A case-control study has reported that the prevalence of suicidal ideation in individuals with MDD ranges from 47 to 69%, with a substantially increased risk of suicide compared to the general population [4]. Given the significant prevalence and detrimental consequences of suicidal ideation in individuals with depression, identifying risk factors associated with this phenomenon is crucial for effective suicide prevention and early intervention efforts.

Previous studies have highlighted the strong association between impulsivity and suicidal ideation and behaviors. Heightened impulsivity is a key predictor of suicidal ideation and is often accompanied by risk-taking behaviors. Individuals with higher impulsivity levels who have attempted suicide are at a greater risk of committing suicide [5, 6]. A recent multivariate regression analysis has indicated that impulsivity remains a significant predictor of suicidal ideation even after accounting for sociodemographic factors, treatment duration, and comorbid depression [7]. Neuroimaging research has also demonstrated a positive correlation between impulsivity, particularly in decision-making processes, and the severity of suicidal ideation in individuals with MDD [8]. Moreover, findings from event-related potential studies have highlighted the relationship between impulsivity, suicidal ideation, and hopelessness [9]. However, the specific psychological mechanisms that connect impulsivity to suicidal ideation remain unknown.

It is well-established that depression and hopelessness are significant risk factors for suicidal ideation [10–12]. Depression is frequently reported as the most common mental illness among individuals who die by suicide, with approximately 60% of those with a current major depressive episode experiencing suicidal thoughts [13, 14]. The severity of depression has been identified as a crucial predictor of both suicidal ideation and behaviors. Similarly, hopelessness has been found to be a central factor in predicting suicide. In a cohort study spanning three decades, hopelessness was clinically shown to predict significant suicidal ideation (odds ratio [OR] = 2.8) among patients with depressive disorders [15]. Another meta-analysis of longitudinal studies found that hopelessness was the strongest predictor of suicide death (weighted odds ratio [wOR] = 1.98) [14]. Additionally, there is evidence from other researchers suggesting that the combination of depression and impulsivity, along with reduced motivational control, can make individuals more vulnerable to suicidal ideation. Furthermore, the interaction between impulsivity and hopelessness has been found to further increase the risk of suicide [16, 17]. Considering the strong relationship observed between impulsivity, depression, hopelessness, and suicidal ideation, it is plausible that depression and hopelessness act as key mediators in the link between impulsivity and suicidal ideation. If this is the case, the present study would contribute to a better understanding of the causal relationship between impulsivity and suicidal ideation.

According to the stress-diathesis model of suicide, trait impulsivity has been identified as a significant predictor and diathesis for suicide risk. However, not all individuals with higher impulsivity tend to exhibit suicidal behavior [18, 19]. Individuals with higher impulsivity are more susceptible to experiencing depression and hopelessness due to their difficulty in controlling negative thoughts. When they are in a state of depressive and hopeless crisis, they are less likely to shift their attention away from thoughts of suicide, which can lead to an increased frequency and continuity of suicidal ideation [20]. Kim et al. [8] suggested that impulsivity, specifically related to reduced motivational control, can influence suicidal ideation in patients with MDD. The combination of depression severity and impulsivity may intensify suicidal ideation. Arango-Tobón et al. [21] also emphasized that impulsivity is a significant risk factor for suicide, particularly in individuals experiencing depressive symptoms and hopelessness. Furthermore, prospective studies have found that depression, mediated by hopelessness, contributes to suicidal ideation [22].

Based on these strong associations and predictor effects, an initial path diagram was constructed among these variables. The proposed model hypothesizes that depression, hopelessness, and impulsivity directly influence suicidal ideation. Impulsivity has a direct effect on both depression and hopelessness. Additionally, impulsivity plays an indirect role in suicidal ideation through its impact on depression and hopelessness.

## Methods

A total of 258 participants were recruited from both outpatients and inpatients with depression who sought treatment at the Weifang Mental Health Center in Shandong province. Inclusion criteria for participants were as follows: (1) aged between 18 and 65 years old, (2) scored on the Hamilton Depression Scale (HAMD-17) by the attending psychiatrist [23] and met the diagnostic criteria for Major Depressive Disorder (MDD) as outlined in the Diagnostic and Statistical Manual of Mental Disorders [24]. Exclusion criteria included patients with a depressive episode in bipolar disorder, depression patients exhibiting current psychotic symptoms, patients currently undergoing modified electroconvulsive therapy (MECT) or transcranial magnetic stimulation therapy (TMS), patients with severe neurological disease or brain trauma, and patients undergoing antidepressant treatment.

All participants were informed that the collected data would be kept confidential and used solely for research purposes. Written informed consent was obtained from each participant. Based on the item on the Scale for Suicide Ideation, participants were divided into two groups: depression patients with suicidal ideation (SI) and depression patients without suicidal ideation (NSI).

### Measures

#### Background variables

In this study, several background variables were investigated based on the existing literature. These variables included age, sex, education level (in years), marital status (single or married), residence identity (city or rural), family history of mental disorders (yes or no), personal history of attempted suicide (yes or no), and presence of physical disease (yes or no).

#### Depression

The Hamilton Depression Scale [23] utilized in this study comprises 17 items, with most items scored on a scale ranging from 0 to 4, while a few items are scored from 0 to 2. The total score on this scale is used to classify the severity of depression into different categories: severe (> 24 points), moderate (17–24 points), mild (7–17 points), and no depressive symptoms (0–7 points). For this study, the Chinese version of the 17-item Hamilton Depression Scale was employed. Previous research conducted with Chinese depression patients reported a Cronbach’s alpha coefficient of 0.71 [24]. In the present study, the Cronbach’s alpha coefficient was found to be 0.78, indicating good internal consistency.

#### Hopelessness

The Chinese Version of the Beck Hopelessness Scale [25] was employed to assess individuals’ negative expectations regarding themselves and the future. The scale consists of 20 items that measure three dimensions: hopelessness for the future, loss of motivation, and expectation for the future. Each item is scored using a binary system, with a score of 1 indicating the presence of hopelessness and a score of 0 indicating its absence. Previous research conducted with Chinese rural suicides reported a Cronbach’s alpha coefficient of 0.92 for the Chinese version of the scale [25]. In the present study, the Cronbach’s alpha coefficient was found to be 0.89, indicating good internal consistency.

#### Suicidal ideation

In the Chinese Version of the Scale for Suicide Ideation [26], there are a total of 19 items. Among these items, items 1–5 are used to assess the presence of suicidal ideation, while items 6–19 measure the intensity of suicidal ideation over the past week. The Cronbach’s alpha coefficient for the rural residents’ sample was 0.57, while for the urban residents’ sample, it was 0.72 [26]. In the present study, the Cronbach’s alpha coefficient was notably higher at 0.95.

#### Impulsivity

The Chinese Version of the Barratt Impulsiveness Scale [27] was used to evaluate individual impulsivity characteristics, including three dimensions of attentional impulsivity, motor impulsivity and no-planning impulsivity. The scale consists of a total of 30 items, which are scored on a 1–5 scale. A higher total score indicates greater impulsiveness. Previous research reported Cronbach’s alpha coefficients ranging from 0.77 to 0.89 for the total scale and subscales of the Barratt Impulsiveness Scale [27]. The test-retest coefficients for the total scale and subscales ranged from 0.68 to 0.89, indicating good reliability over time. In the present study, Cronbach’s alpha coefficient was 0.93.

### Statistical analysis

SPSS version 21 was used for statistical description of the data. Categorical variables of background characteristics were represented by percentages. Quantitative variables of background characteristics and psychological variables with normal distribution were described by means (M) and standard deviations (SD). To compare the background characteristics and psychological variables between the suicidal ideation (SI) group and non-suicidal ideation (NSI) group, t-tests were conducted for quantitative variables, while Chi-square tests were employed for categorical data. Pearson correlation analyses were performed to examine the correlations among the study variables.

Amos 21 software was used to construct structural equation models among depression, hopelessness, impulsivity and suicidal ideation to analyse the mediation path. To estimate the total, direct, and indirect effects, the PROCESS macro for SPSS (model 6) was employed. A total of 5000 bootstrapping re-samples were used to calculate the 95% confidence interval (CI). If the interval did not contain 0, the effects were considered statistically significant [28].

## Results

### Descriptive analyses

As shown in Table 1, there were significant differences in age and the distribution of marital status between the SI group and the NSI group (p < 0.01). The patients in the SI group were younger in age, and a higher proportion of them were married. However, there were no significant differences in the distribution of sex, residence identity, family history of mental disorders, personal history of attempted suicide, and presence of physical disease between the two groups. In addition, there were significant differences in the scores of depression, suicidal ideation, hopelessness, and impulsivity between the SI group and the NSI group, and the scores of these variables in SI group were significantly higher than those in NSI group.

**Table 1:** Comparisons between the SI and NSI group in background characteristics and psychological variables

Characteristics		SI n(%)	NSI n(%)	χ^2^/*t* test	*p*
Age		44.80 ± 14.85	52.28 ± 10.82	-4.42	< 0.01
Education level (in years)		8.53 ± 3.24	8.97 ± 4.26	-0.90	0.37
Sex	Male	48(31.4)	30(28.6)	0.23	0.63
	Female	105(68.6)	75(71.4)		
Residence identity	Rural	66(43.1)	51(48.6)	0.74	0.39
	City	87(56.9)	54(51.4)		
Marital status	Single	21(13.7)	33(31.4)	11.20	< 0.01
	Married	132(86.3)	72(68.6)		
Family history of mental disorders	Yes	12(7.8)	15(14.3)	2.76	0.10
	No	141(92.2)	90(85.7)		
Personal history of attempted suicide	Yes	6(3.9)	3(2.9)	0.21	0.65
	No	147(96.1)	102(97.1)		
Presence of physical disease	Yes	30(19.6)	24(22.9)	0.40	0.53
	No	123(80.4)	81(77.1)		
Depression		23.88 ± 6.64	15.86 ± 5.74	10.07	< 0.01
Suicidal ideation		36.10 ± 8.93	17.54 ± 5.49	20.64	< 0.01
Hopelessness		14.31 ± 4.92	8.14 ± 5.39	9.52	< 0.01
Impulsivity		90.22 ± 23.07	78.66 ± 19.34	4.36	< 0.01

### Partial correlation analysis

Table 2 presents the results of the partial correlation analysis conducted among the psychological variables in depression patients, while controlling for age and marital status. The analysis revealed a strong relationship between suicidal ideation and the following variables: depression (r = 0.687), hopelessness (r = 0.694), and impulsiveness (r = 0.387).

**Table 2:** Partial correlation analysis among psychological variables in depression patients

	Depression	Suicidal ideation	Hopelessness	Impulsivity
Depression	1			
Suicidal ideation	0.687**	1		
Hopelessness	0.591**	0.694**	1	
Impulsivity	0.399**	0.387**	0.403**	1

(** *p* < 0.01)

### Path analysis

**Fig. 1:**
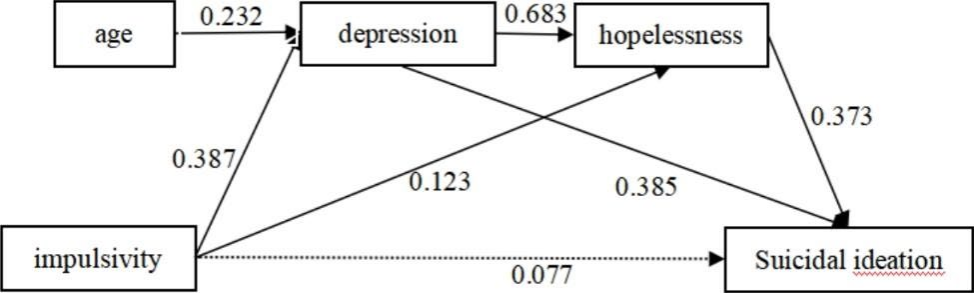
Path analysis of impulsivity, depression, hopelessness, and suicidal ideation

The proposed model was tested by including all the variables, and it demonstrated a good fit to the data (χ2 = 1.135, p = 0.769; RMSEA = 0.000; GFI = 0.998; AGFI = 0.991). As depicted in Table 3; Fig. 1, the results indicated significant direct effects. Specifically, there was a significant direct effect of impulsivity on both depression (β = 0.387, p < 0.001) and hopelessness (β = 0.123, p < 0.001). Similarly, depression had a significant direct effect on both hopelessness (β = 0.683, p < 0.001) and suicidal ideation (β = 0.385, p < 0.001). Additionally, hopelessness demonstrated a significant direct effect on suicidal ideation (β = 0.373, p < 0.001). However, the direct effect of impulsivity on suicidal ideation was not found to be significant (β = 0.077, p = 0.083).

**Table 3:** Regression analysis between variables

Dependent variable	Predictor variables	β	Bootstrapping 95% CI		*p*
			LLCI	ULCI	
Depression	Age	0.232	0.086	0.310	< 0.001
	Marital status	0.024	-2.943	4.235	0.723
	impulsivity	0.387	0.139	0.249	< 0.001
Hopelessness	Age	0.117	0.009	0.097	0.019
	Marital status	-0.060	-2.261	0.508	0.214
	impulsivity	0.123	0.010	0.056	< 0.01
	depression	0.683	0.315	0.411	< 0.001
Suicidal ideation	Age	0.079	-0.017	0.161	0.110
	Marital status	0.025	-2.035	3.482	0.606
	impulsivity	0.077	-0.005	0.088	0.083
	depression	0.385	0.282	0.542	< 0.001
	hopelessness	0.373	0.504	0.996	< 0.001

According to the results presented in Table 4, several mediation effects were observed in the relationship between impulsivity, depression, hopelessness, and suicidal ideation. Firstly, depression was found to mediate the relationship between impulsivity and suicidal ideation. The mediating effect of depression accounted for 40.26% of the total effect. Secondly, hopelessness was found to mediate the relationship between impulsivity and suicidal ideation. The mediating effect of hopelessness accounted for 12.41% of the total effect. Furthermore, a serial mediation effect was observed, where depression and hopelessness serially mediated the relationship between impulsivity and suicidal ideation. The mediating effect of depression and hopelessness accounted for 26.59% of the total effect.

**Table 4:** Mediating effects of depression and hopelessness on the relationship between impulsivity and suicidal ideation

	Effect	Boot SE	BootLLCI	BootULCI	Relative mediating effect value
Total indirect effect	0.294	0.042	0.211	0.372	79.31%
Ind1: impulsivity->depression->Suicidal ideation	0.149	0.032	0.093	0.216	40.26%
Ind2: impulsivity->hopelessness->Suicidal ideation	0.046	0.018	0.013	0.083	12.41%
Ind3:impulsivity->depression->hopelessness->Suicidal ideation	0.099	0.021	0.060	0.142	26.59%

## Discussion

This study not only provides insights into the prevalence of suicidal ideation among depressed patients but also sheds light on the relationship between depression, hopelessness, impulsivity, and suicidal ideation using a serial multiple mediation model. The findings indicate that the prevalence of suicidal ideation among depressed patients in this study was 59.3%, indicating a higher likelihood of suicidal ideation in individuals with depression. Suicidal ideation, as an early indicator of suicidal thoughts, reflects the negative thinking and intense inner pain experienced by individuals at that time, and it serves as an important risk factor for suicidal behavior [29]. Given the strong association between suicidal ideation and suicidal behavior in depressed patients, it is crucial for clinicians to inquire about and assess the presence of suicidal ideation when obtaining the medical history and conducting routine psychiatric examinations [30].

Extensive research has consistently demonstrated that depression is a significant risk factor for suicidal ideation, and the severity of depression is closely related to an increased incidence and intensification of suicidal ideation [31, 32]. The results of this study also revealed that the severity of depression significantly predicted suicidal ideation, with the depressed group with suicidal ideation exhibiting more severe depression compared to the depressed group without suicidal ideation. Typically, individuals in a depressed state tend to interpret, think, and react to events in a more negative and despairing manner. The severity of depression can exacerbate this tendency, making depressed individuals more vulnerable to persistent depressive symptoms and negative moods. Consequently, these vulnerable patients are at an elevated risk of experiencing suicidal thoughts and may even contemplate ending their lives [33, 34]. Furthermore, it is well-established that interventions targeting the reduction of depression severity can lead to a decrease in subsequent suicidal thinking or attempts [35]. Therefore, psychiatrists should give serious consideration to the issue of suicide, inform the families of patients to provide appropriate care, and take preventive measures to mitigate the occurrence of suicide when dealing with patients presenting with severe depressive symptoms.

Meanwhile, hopelessness has consistently been identified as a strong predictor of suicide in individuals with depression [36, 37]. It is a characteristic feature of depressive symptoms and is commonly observed in patients with depression. The present study also found a significant association between hopelessness, suicidal ideation, and depression, with hopelessness demonstrating a significant predictive effect on suicidal ideation. Hopelessness reflects an individual’s negative expectations about the future, which stems from a fixed negative thinking pattern formed by dysfunctional attitudes [37]. This negative cognitive pattern remains hidden behind feelings of pessimism and hopelessness. When individuals are consumed by negative expectations and pessimism, they firmly believe that their current pain and difficulties will persist indefinitely, and they perceive the future as being devoid of relief from hardships, setbacks, and losses. Consequently, they experience feelings of hopelessness, helplessness, and worthlessness [38].

In addition, a considerable body of research has consistently demonstrated a strong association between impulsivity, hopelessness, and suicide [39, 40]. Studies have shown that individuals with higher levels of impulsivity are more likely to experience suicidal ideation [41]. Consistent with these findings, the present study also found a significant difference in the total impulsivity scores between the depressed group with suicidal ideation and the depressed group without suicidal ideation, with higher impulsivity scores observed in the former group. This suggests that impulsivity is a risk factor for suicidal ideation in individuals with depression. Dumais et al. proposed that individuals with high levels of impulsivity may struggle to manage and control their thoughts effectively. They may have difficulty suppressing irrational thoughts, which can contribute to the development of suicidal ideation [41]. This aligns with the notion that impulsivity can influence cognitive processes and decision-making, thereby increasing the risk of suicidal ideation.

The perspective put forth by Aaltonen et al. [36] regarding the need for intermediary factors to demonstrate the effects of certain factors in the presence of interactions among various factors is relevant to this study. Consistent with this view, the results of the serial multiple mediation model showed significant positive correlations between impulsivity, depression, and hopelessness. Moreover, the findings indicated that impulsivity indirectly contributes to suicidal ideation through its influence on depression and hopelessness. It is plausible that depressed patients with impulsive personality traits may exhibit irrational beliefs, inflexible thinking patterns, and poor problem-solving abilities. When faced with setbacks in life, depressive rumination may hinder the successful retrieval of positive memories and increase the likelihood of negative interpretations of events [42]. Consequently, these individuals may struggle to envision positive aspects of the future and lose hope, leading to feelings of depression, pessimism, and despair. Simultaneously, individuals with high impulsivity, due to their lack of self-control and rationality, may be prone to impulsive and unplanned reactions when confronted with problems. They may fail to consider the potential negative consequences of their actions on themselves or others. In such cases, suicide may be seen as a way to escape or solve their problems [43].

Considering the implications of these findings, it is crucial to prioritize the identification and support of depressed patients who exhibit high levels of impulsivity and hopelessness. This targeted approach can be an important strategy in suicide prevention efforts. By addressing impulsivity and hopelessness in addition to depression, interventions can potentially help individuals develop better coping mechanisms, enhance problem-solving skills, and foster a sense of hope for the future.

The present study has several limitations that should be acknowledged. Firstly, the participants were selected using convenience sampling from depressed patients at Weifang Mental Health Center, which may introduce regional biases and limit the generalizability of the findings. Therefore, caution should be exercised when drawing conclusions and making recommendations based on these findings. Secondly, this study employed a cross-sectional design, which limits its ability to establish causal relationships. Longitudinal studies would be valuable in confirming the causal effects of the associations observed in this study and providing a more robust understanding of the predictive power for suicide in depressed patients. Lastly, while this study demonstrated that controlling impulsivity indirectly reduces suicidal ideation, it remains unclear whether impulsivity is solely linked to the risk of suicidal ideation or if it is also associated with suicide attempts. Further research should consider stratifying the suicidal ideation group into those with suicidal ideation alone and those with suicidal ideation accompanied by suicide attempts. This stratification would help to address the question of whether impulsivity is related to both suicidal ideation and suicide attempts. Overall, these limitations highlight the need for future studies to employ more diverse and representative samples, utilize longitudinal designs, and investigate the specific associations between impulsivity, suicidal ideation, and suicide attempts in order to enhance our understanding of these complex relationships.

## Conclusions

This study investigated the relationship between impulsivity, depression, hopelessness, and suicidal ideation. The findings revealed a serial multiple mediation effect, indicating that depression and hopelessness mediate the pathway from impulsivity to suicidal ideation. Specifically, depression was found to mediate the association between impulsivity and suicidal ideation, and hopelessness further mediated the relationship between depression and suicidal ideation. Based on these results, it is recommended that interventions targeting impulsivity, depression, and hopelessness should be strengthened in order to effectively prevent suicide among individuals who are experiencing depression. By addressing these factors, it may be possible to reduce the risk of suicidal ideation and ultimately prevent suicide in depressed patients.

## Data Availability

The datasets used or analysed during the current study are available from the corresponding author on reasonable request.

## Abbreviations

MDD: Major depressive disorder
SI: Depression patients with suicidal ideation group
NSI: Depression patients without suicidal ideation group
CI: Confidence interval
ULCI: Upper level of confidence interval
LLCI: Lower level of confidence interval
